# Development and Psychometric Validation of the Brief Nurses' Practice Environment Scale and Its Relation to Burnout Syndrome and Job Satisfaction: A Study in Spanish Nurses

**DOI:** 10.3389/fpubh.2021.621991

**Published:** 2021-08-17

**Authors:** Noemí Sansó, Gabriel Vidal-Blanco, Laura Galiana, Amparo Oliver

**Affiliations:** ^1^Department of Nursing and Physiotherapy, University of the Balearic Islands, Palma de Mallorca, Spain; ^2^Balearic Islands Health Research Institute (IdISBa), Palma de Mallorca, Spain; ^3^Department of Nursing, University of Valencia, Valencia, Spain; ^4^Department of Methodology for the Behavioral Sciences, University of Valencia, Valencia, Spain

**Keywords:** instrument development (MeSH), burnout - professional, practice nursing, factor analisys, job satisfaction

## Abstract

**Introduction:** Nursing environment is a vast concept that traditionally has included a wide range of job characteristics and has been related to burnout and job satisfaction. For its measurement, the Practice Environment Scale of the Nursing Work Index (PESNWI) stands out. However, shorter instruments are needed. The purpose of the study is to develop and test the Brief Nurses' Practice Environment (BNPE) Scale.

**Methods:** The BNPE Scale was developed and tested in a sample of 210 Spanish nurses (data collection 2018).

**Results:** Cronbach's alpha was 0.702. The confirmatory factor analysis (CFA), with an excellent fit, offered evidence of internal validity. Regarding validity, the BNPE Scale predicted both burnout and job satisfaction. Finally, evidence pointed out a cutoff score of <12 for low levels of practice environment and a cutoff score of >15 for higher levels in practice environment.

**Conclusions:** The BNPE Scale is a short, easy-to-use measure that could be employed in major batteries assessing the quality of healthcare institutions.

## Introduction

Nursing environment is a vast concept that traditionally has included a wide range of job characteristics and has been defined as the organizational characteristics in the work environment that make the professional practice easier or more difficult, and it is considered favorable when the nurses have autonomy, control over the work environment, and good relationships with the health team (1). As such, the study of nursing environment has been traditionally carried out with two different but complementary approaches: the psychosocial and the organizational one (2–4). The first has focused on psychological and relational aspects related to the workplace, whereas the latter has pointed the importance of job characteristics in defining perceptions of nurses about work environment. However, and as already pointed, these are not opposite but complementary parts of working environment of nurses, and they tend to interact between them (5). According to the American Organization for Nursing Leadership (6), the key elements of a healthy practice environment are (1) a collaborative practice culture, (2) a communication-rich culture, open and trusty, (3) a culture of accountability in which role expectations are clearly defined and everyone is accountable, (4) the presence of adequate numbers of qualified nurses, (5) the presence of competent leadership in which leaders support shared decision-making and allocate resources to support nursing, (6) shared decision-making at all levels, (7) the encouragement of professional practice and continued development, (8) recognition of the value of contribution of nursing, and (9) recognition by nurses for their meaningful contribution to practice. In summary, a healthy nursing environment is a workplace that is safe, empowering, and satisfying (7).

Within this framework, much efforts have been done to detect and improve work environment psychosocial and organizational factors that can be sources of burnout and job satisfaction, so that to improve work organization (8).

Regarding burnout of nurses, there is a vast body of research that has pointed how working conditions, such as low job control and high job demands, being moved among different patient care units within the organization, being short of essential resources, and having low supportive work relationships with co-workers, supervisors, and/or physicians, may produce higher levels of burnout (9–14). Indeed, a recent review (15) has pointed avoidance of conflict management style (16), low job satisfaction (17), and the increased nurse-patient ratios (18) as burnout facilitators. As regards job satisfaction, it is influenced by both work psychosocial and organizational factors (12, 19). Consequently, working environment characteristics of nurses, such as autonomy or nurse–physician collaboration, are clue for nurses to maintain an adequate degree of affect toward a job and its main components (19–22).

Because of its relation to burnout and job satisfaction of nurses, interest of researchers and managers on identifying the specific factors conforming practice environment of nurses has been stated, and many efforts on its measurement have been done in the scientific literature. Gershon et al. (23), for example, identified 12 instruments design to study culture, climate, and work environment of nurses. Some of them include, for example, the Dutch Essentials of Magnetism II instrument, recently designed to assess nursing practice environments (24). Among the tools developed to assess work conditions of nurses, one of the first developed and internationally well-known indexes is the Nursing Work Index, originally designed by Kramer and Hafner (25). This scale included 65 items in its original version, but shortened versions with 57 (26) and 31 items (1) have been developed and widely used. The 31-item version developed by Lake, the Practice Environment Scale of Nursing Work Index (PESNWI), is one of the most used scales, with low respondent burden, adequate psychometric properties, and high discriminant ability (1, 27).

The PESNWI evaluates those factors in the work environment of nurses that may enhance or interfere with abilities of nurses to provide care, and it has grouped them into five dimensions: (1) nurse participation in hospital affairs; (2) nursing foundations for quality care; (3) nurse manager leadership, ability, and support; (4) adequate staffing and resources; and (5) collegial nurse–physician relationships (1, 5, 28, 29). Higher scores on the PESNWI have been associated with burnout (12, 22, 30), job satisfaction (12), better quality of care (31, 32), as well as better patient-reported experiences of care (33).

Therefore, the measurement of nursing practice environment is clue for health managers and institutions. However, conditions and outcomes to measure when working in the healthcare context are enormous, and having to answer so many questionnaires makes the workload of the nurses even greater, shortening their valuable time. For these reasons, it is very important to have short tools that allow us to screen and detect potential problems. And then, only then, apply the longer batteries in order to better determine and understand the conditions that aim to be solved. In this line of thought, and from a preventive point of view, a brief screening tool will serve also to periodically assess the several outcomes and dimensions of the healthcare system.

### Purpose

The aim of this study was to develop and test the psychometric properties of the Brief Nurses' Practice Environment (BNPE) Scale for the Spanish population, a short measure of nursing practice environment, based on the traditional dimensions identified as important for maintaining adequate levels of stress, job satisfaction, and quality of patient care. For that purpose, a brief scale, based on the PESNWI, was developed, presented, and validated.

## Methods

### Design, Setting, and Participants

The study had a cross-sectional design with a correlational approach. Data were gathered at one time point, during the months of June, July, and August of 2018. First, nursing managers of the health centers on the Balearic Islands were invited to participate, initially through a written letter and then through personal interviews where the research project was explained in detail. Once their permission had been obtained and the Ethical Research Committee of the University of the Balearic Islands approved the project, each nursing manager was asked to send the invitation letter to the nurses in the center. This invitation was sent by mail with the link to the survey, which was hosted on an online platform. On this same platform, the participating nurses signed the informed consent. Confidentiality of the data was ensured.

Sample size was estimated following Wolf et al.'s work (34), in which the authors carried out Monte Carlo data simulation techniques to evaluate sample size requirements for common applied structural equation modeling. Taking into account that the confirmatory factor analysis (CFA) was a one-factor, five-indicator model, but loadings were expected to be between 0.30 and 0.80, we took the most conservative data of 190 (34). Assuming there could be participants with missing data in all the variables, we increased the sample size up to 210 participants.

Inclusion criteria: Participants were nurses working in the Healthcare Public System of the Balearic Islands at the moment of the study, including hospitals and primary healthcare centers. Regarding hospitals, there were two of them dedicated to the treatment of chronic disease. Exclusion criteria: Those nurses not working in the moment of the survey or working exclusively in administration tasks (not developing care activity) were excluded in order to address potential sources of bias.

### Measures

The Brief Nurse's Practice Environment Scale is based on the original PESNWI dimensions, which included (1) nurse participation in hospital affairs; (2) nursing foundations for quality care; (3) nurse manager leadership, ability, and support; (4) adequate staffing and resources; and (5) collegial nurse–physician relationships. Taking these concepts as the key points of the practice nursing environment, two experts in nursing and psychometrics turned the dimensions into the five final items that composed the BNPE. The BNPE is composed of five sentences, representing the five dimensions of the PESNWI. The sentences were rated according to agreement, using a Likert-type, 4-point scale, from 1 (completely disagree) to 4 (completely agree). Total score was calculated by summing the scores in the five items and ranged from 5 to 20. Item content can be consulted in Table 1.

**Table 1:** The brief nurses' practice environment scale (BNPE scale).

**Instructions: mark the degree to which you agree with the following statements regarding your work environment. Take into account that 1 implies that you totally disagree with the proposed sentence and 4 means that you completely agree with the statement**.						**M**	**SD**	***α_IID_***	**H**
**Item num**.	**Item content**	**Completely** ** disagree**	**Disagree**	**Agree**	**Completely** ** agree**				
Item 1	Nurses participate in hospital affairs.	1	2	3	4	2.76	0.851	0.582	0.597
Item 2	Care is based on nursing foundations.	1	2	3	4	3.29	0.682	0.379	0.683
Item 3	Nurse manager shows leadership, ability, and support to nurses.	1	2	3	4	2.54	0.953	0.622	0.572
Item 4	Staff and resources are adequate.	1	2	3	4	2.06	0.889	0.334	0.708
Item 5	Collegial nurse-physician relationships are adequate.	1	2	3	4	2.49	0.722	0.397	0.677

*M, mean; SD, standard deviation; α_iid_, alpha if item deleted; H, item homogeneity*.

Two additional scales were also used: the short 9-item version of the Maslach Burnout Inventory (MBI) (35) and the General Work Satisfaction Scale from the Michigan Organizational Assessment Scale (36). Internal consistency estimates were 0.808 and 0.723, respectively.

### Ethical Considerations

The study was approved by the Ethical Research Committee of the University of the Balearic Islands (82CER18). People who decided to participate voluntarily were told the reason and purpose for carrying out the study. The study was carried out in compliance with the ethical principles for research in health sciences established in the Declaration of Helsinki—Ethical principles for medical research involving human subjects (37). Anonymity, confidentiality, and protection of privacy were guaranteed. Participants voluntarily signed an informed consent authorizing the collection and processing of the information, and they were able to withdraw their consent at any time and without any consequences.

### Analysis

Analyses included descriptive statistics and estimates of reliability, including Cronbach's alpha, item homogeneity, and alpha if item deleted.

As regards internal validity, and in order to study the factorial structure of the Brief PESNWI, a one-factor CFA model was tested. Evidence of external validity was gathered by studying the relation between BNPE and burnout and job satisfaction. A structural equation model (SEM) was tested, in which practice environment predicted the three dimensions of burnout, and these, together with the practice environment, predicted job satisfaction.

Fit of models was assessed using the following statistic and fit indexes: the chi-square, the comparative fit index (CFI), and the root mean-squared error of approximation (RMSEA). Adequate fit is generally assumed with CFI > 0.90 together with a RMSEA < 0.08, while values of CFI > 0.95 and RMSEA < 0.06 indicate an excellent fit (38). The method of estimation of both models was weighted least-squares mean and variance-corrected (WLSMV) (39).

Finally, in order to offer easy interpretation of the scale results when used by managers, cutoff criteria were offered. Quartiles were calculated, and those values corresponding to quartile 1 (lowest 25% of the sample) and quartile 3 (highest 25% of the sample) were chosen as cutoff criteria to classify participants into low, medium, and high practice environment levels. Participants were grouped following these cutoff points, and the distribution across gender were studied, and their means on age, years in nursing, years in current area/specialty, years in current job position, and the dimensions of burnout and job satisfaction were compared. Differences across genders were studied using chi square; differences across age, years in nursing, years in current area/specialty, and years in current job position were studied using ANOVA; a MANOVA studying the effect of the level of practice environment on job burnout was carried out; and also an ANOVA was conducted with job satisfaction as the dependent variable.

## Results

Two hundred and ten nurses answered the survey, working in 14 different centers on the Balearic Islands, including hospitals. One hundred and fifty-eight were women. Mean age was 40.24 years old. Characteristics of the sample are displayed in Table 2.

**Table 2:** Characteristics of the sample.

**Variables**		**M**	**SD**
Age		40.24	9.78
Years in nursing		3.75	2.05
Years in current area/specialty		2.40	1.74
Years in current job position		1.86	1.49
**Variables**	**Categories**	**N**	**%**
Gender	Women	158	75.2
	Men	29	13.8
	Missing data	23	11.0
Shifts	Without shifts	97	46.2
	With shifts	88	41.9
	Missing data	25	11.9
Working day duration	8 h	153	72.9
	10 h	5	2.4
	12 h	24	11.4
	Missing data	28	13.3
Job situation	Public worker	119	56.7
	Acting official	29	13.8
	Temporary worker	39	18.6
	Missing data	23	11.0

The study of the reliability estimates of the scores pointed adequate results. Estimate of the internal consistency of the scale by Cronbach's alpha was 0.702, and items showed adequate homogeneity and reliability (Table 1).

The CFA showed an excellent overall fit: $\chi_{\left(5\right)}^{2}$ = 8.627 (*p* = 0.124), CFI = 0.987, and RMSEA = 0.065 (0.000, 0.136). All factor loadings were statistically significant (*p* < 0.001) (Table 1).

**Figure 1:**
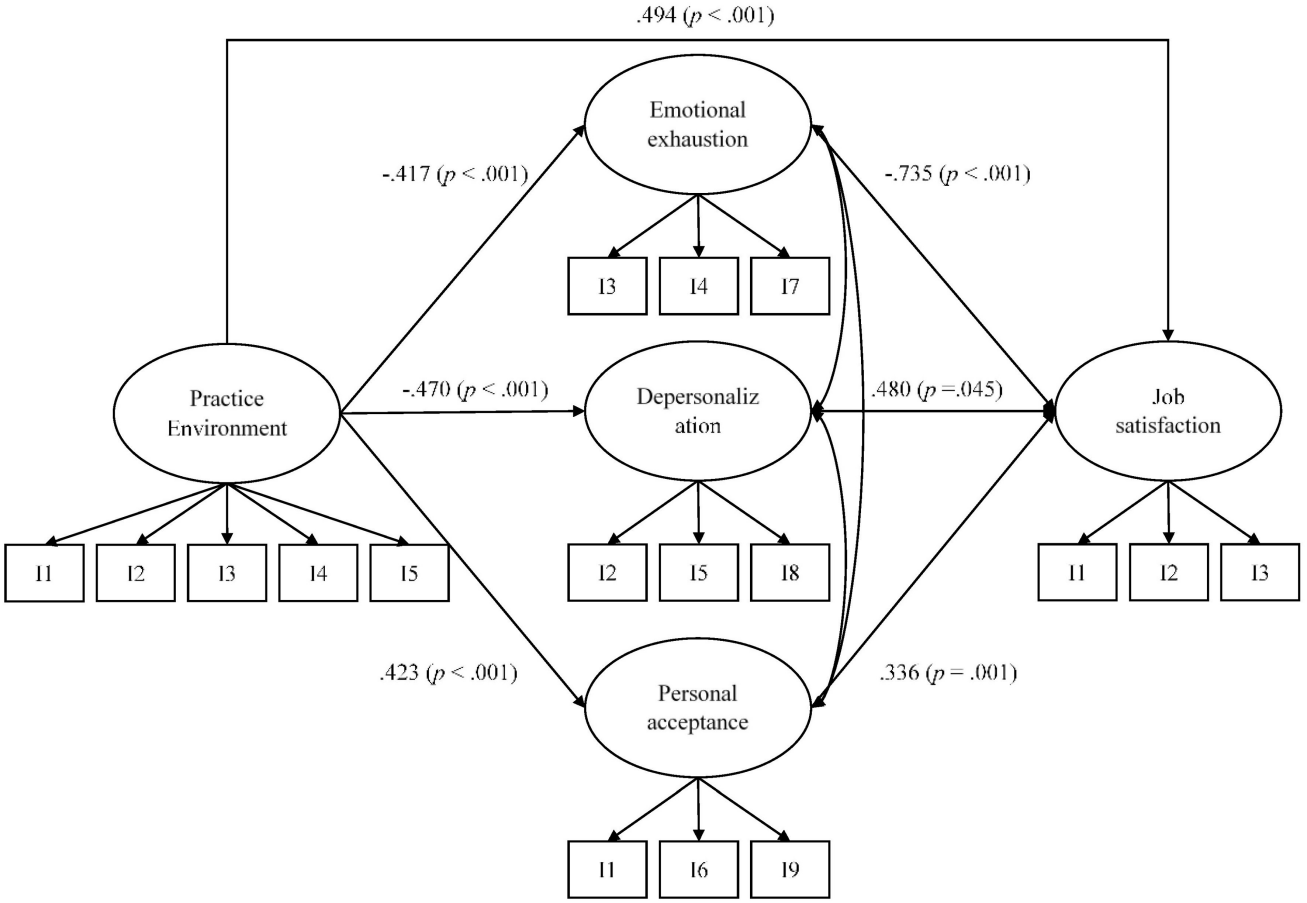
Results of the predictive model with the BNPE scale. For the sake of clarity, only predictive relations are shown. All factor loadings were statistically significant (*p* < 0.001). Correlations among the burnout dimensions were: 0.768 (*p* < 0.001) between emotional exhaustion and depersonalization; −0.315 (*p* < 0.001) between emotional exhaustion and personal acceptance; and −0.486 (*p* < 0.001) between depersonalization and personal acceptance.

The SEM in which practice environment predicted burnout and job satisfaction showed an adequate overall fit: $\chi_{\left(109\right)}^{2}$ = 243.855 (*p* < 0.001), CFI = 0.940, and RMSEA = 0.085 (0.071, 0.099). Results pointed adequate predictive power of practice environment on the dimensions of both burnout syndrome and job satisfaction, being all the proposed relations statistically significant (*p* < 0.001) and in the expected direction: Higher levels of practice environment, as measured with the BNPE scale, predicted higher scores in personal acceptance and job satisfaction, whereas lower levels predicted higher scores in emotional exhaustion and depersonalization (Figure 1).

Finally, cutoff criteria were offered. Quartiles were calculated, and participants were classified into low (< 12), medium (12–15), and high (>15) practice environment levels and their means in sociodemographic characteristics and burnout and job satisfaction were compared.

Regarding the relation between practice environment levels and sociodemographic characteristics, no statistically significant relation was found with sex [${\text{χ}}_{\left(2\right)}^{2}$ = 1.564, *p* = 0.458, Cramer's V = 0.096], age [*F*_(2, 163)_ = 0.485, *p* = 0.617, η^2^= 0.006], years in nursing [*F*_(2, 168)_ = 0.328, *p* = 0.721, η^2^= 0.004], years in current area/specialty [*F*_(2, 167)_ = 0.187, *p* = 0.829, η^2^= 0.002], or years in current job position [*F*_(2, 167)_ = 0.137, *p* = 0.872, η^2^= 0.002].

The MANOVA studying the effect of the level of practice environment on job burnout showed statistically significant differences between the means in burnout: *F*_(6, 334)_ = 6.136, *p* = 0.001, η^2^= 0.063. Follow-up ANOVAs showed statistically significant differences applying Bonferroni corrections between groups in emotional exhaustion [*F*_(2, 168)_ = 9.798, *p* < 0.001, η^2^= 0.104], depersonalization [*F*_(2, 168)_ = 3.533, *p* = 0.031, η^2^= 0.040], and personal acceptance [*F*_(2, 168)_ = 4.192, *p* = 0.017, η^2^= 0.048]. *Post-hoc* tests showed statistically significant mean differences in emotional exhaustion between low and medium (*p* = 0.004) and between low and high (*p* < 0.001) practice environment, with higher levels of emotional exhaustion for those nursing working in places with lower levels of practice environment. That is, the relation between emotional exhaustion and practice environment was negative: The lower the practice environment, the higher the emotional exhaustion. In depersonalization, *post-hoc* differences were found between those with low and those with high levels of practice environment (*p* = 0.024), with higher levels of depersonalization in nurses with lower levels of practice environment. Again, the lower the practice environment, the higher the depersonalization. And finally, in personal accomplishment, differences were found between those with low and high levels of practice environment, with higher levels of personal accomplishment for those nurses with higher levels of practice environment (*p* = 0.013). Thus, the relation between personal accomplishment and practice environment was positive: The higher the practice environment, the higher the personal accomplishment.

The ANOVA studying the effect of practice environment on mean scores of job satisfaction also resulted statistically significant difference: *F*_(2, 168)_ = 9.161, *p* < 0.001, η^2^= 0.098. *Post-hoc* statistically significant differences in job satisfaction were found between low vs. medium levels of practice environment (*p* = 0.011), and low vs. high levels of practice environment (*p* < 0.001). The higher the practice environment level, the higher the job satisfaction. Descriptive statistics are presented in Table 3.

**Table 3:** Descriptive statistics of practice environment, burnout dimensions and job satisfaction of the groups with low, medium and high levels of practice environment.

	**Practice environment**		**Burnout dimensions**						**Job satisfaction**	
			**Emotional exhaustion**		**Depersonalization**		**Personal accomplishment**		
	**M**	**SD**	**M**	**SD**	**M**	**SD**	**M**	**SD**	**M**	**SD**
Low PE (<12)	9.32	1.54	8.70	4.44	5.02	3.87	11.45	3.20	8.97	2.01
Medium PE (12–15)	13.47	1.12	6.12	4.26	4.26	3.24	12.68	3.14	9.86	1.56
High PE (>15)	17.13	1.01	4.30	3.83	2.90	2.80	13.57	2.85	10.63	1.19

*PE, Practice environment; M, Mean; SD, Standard deviation*.

## Discussion

The aim of the study was to develop and test the psychometric properties of the BNPE Scale, a short measure of nursing practice environment. The scale, composed by five items that represented the five original dimensions of the PESNWI, presented adequate evidence of reliability and both internal and external validity in a sample of Spanish nurses. Also, information for the interpretability and usage of the instrument was gathered.

Reliability was assessed, with also good results, at the level of the scale and also at the item level. Also, our results pointed estimates of adequate internal validity, supporting the appropriateness of the one-factor structure of the BNPE scale. Its five items, named after the PESNWI dimensions, loaded into a single dimension of practice environment of nurses. The practice environment of nurses, as measured with the BNPE, showed evidence of test criterion validity in the context of a SEM, being a strong predictor of both burnout syndrome and job satisfaction. This is in line with previous research, which had already pointed how bad conditions in working environment of nurses can produce high levels of burnout (9–18), whereas, when adequate, working environment of nurses can lead to high levels of job satisfaction (12, 19, 20).

Finally, results regarding interpretability provided evidence for a cutoff score of <12 for detecting problems in working conditions of nurses (low levels of practice environments), whereas a cutoff score of >15 was proposed and adequately worked for detecting higher levels in practice environment. These criteria were found to be useful in detecting nurses with high levels of burnout and problems with job satisfaction, specifically with regard to those that showed low levels of practice environment.

Limitations of the study are mainly referred to the characteristics of the sample, which was not big and limited to the Spanish context. This could affect the generalizability of the results. As we did not gather information on ward distribution, family status, or type of patients with whom the work activities are carried out, the relation of practice environment with these variables could not be studied. Also the briefness of the scale, although one of its main strengths, could be seen as a limitation. Capturing complex realities such as nursing working environment with a small group of items is difficult, and thus, we would like to highlight again the screening purpose of the scale, offering then a tool to detect problems in the working environment, but not to provide a specific diagnostic of them.

Based on the well-known and widely recognized model of the PESNWI, the BNPE scale is a short, easy-to-use measure that could be employed in major batteries assessing quality of Spanish healthcare institutions, with cutoff points for indicating the presence of practice environment problems and probably high levels of burnout and low job satisfaction. In such cases, deeper studies, with longer scales and personal interviews, are recommended.

## Data Availability Statement

The raw data supporting the conclusions of this article will be made available by the authors, without undue reservation.

## Ethics Statement

The study was reviewed and approved by the Ethical Research Committee of the University of the Balearic Islands (Reference number: 82CER18).

## Author Contributions

LG: conceptualization, methodology, formal analysis, and writing—original draft. GV-B: investigation and writing—review and editing. AO: formal analysis and writing—review and editing. NS: conceptualization, methodology, investigation, writing—review and editing, and project administration. All authors contributed to the article and approved the submitted version.

## Conflict of Interest

The authors declare that the research was conducted in the absence of any commercial or financial relationships that could be construed as a potential conflict of interest.

## Publisher's Note

All claims expressed in this article are solely those of the authors and do not necessarily represent those of their affiliated organizations, or those of the publisher, the editors and the reviewers. Any product that may be evaluated in this article, or claim that may be made by its manufacturer, is not guaranteed or endorsed by the publisher.
